# Coevolution of COVID-19 research and China’s policies

**DOI:** 10.1186/s12961-021-00770-6

**Published:** 2021-09-06

**Authors:** Xi Cheng, Li Tang, Maotian Zhou, Guoyan Wang

**Affiliations:** 1 Department of Digital Communication, Soochow University, Room 5146, Building 1005, No.1 Wenjing Road, Dushu Lake Campus of Soochow University, Suzhou, Jiangsu China; 2 Department of Public Administration, Fudan University, Shanghai, China; 3 School of Medicine, Emory University, Atlanta, USA

**Keywords:** Science and policy, Coevolution model, COVID-19, China

## Abstract

**Background:**

In the era of evidence-based policy-making (EBPM), scientific outputs and public policy should engage with each other in a more interactive and coherent way. Notably, this is becoming increasingly critical in preparing for public health emergencies.

**Methods:**

To explore the coevolution dynamics between science and policy (SAP), this study explored the changes in, and development of, COVID-19 research in the early period of the COVID-19 outbreak in China, from 30 December 2019 to 26 June 2020. In this study, VOSviewer was adopted to calculate the link strength of items extracted from scientific publications, and machine learning clustering analysis of scientific publications was carried out to explore dynamic trends in scientific research. Trends in relevant policies that corresponded to changing trends in scientific research were then traced.

**Results:**

The study observes a salient change in research content as follows: an earlier focus on “children and pregnant patients”, “common symptoms”, “nucleic acid test”, and “non-Chinese medicine” was gradually replaced with a focus on “aged patients”, “pregnant patients”, “severe symptoms and asymptomatic infection”, “antibody assay”, and “Chinese medicine”. “Mental health” is persistent throughout China’s COVID-19 research. Further, our research reveals a correlation between the evolution of COVID-19 policies and the dynamic development of COVID-19 research. The average issuance time of relevant COVID-19 policies in China is 8.36 days after the launching of related research.

**Conclusions:**

In the early stage of the outbreak in China, the formulation of research-driven-COVID-19 policies and related scientific research followed a similar dynamic trend, which is clearly a manifestation of a coevolution model (CEM). The results of this study apply more broadly to the formulation of policies during public health emergencies, and provide the foundation for future EBPM research.

**Supplementary Information:**

The online version contains supplementary material available at 10.1186/s12961-021-00770-6.

*Laws and institutions must go hand in hand with the progress of the human mind. As that becomes more developed, more enlightened, as new discoveries are made, new truths discovered and manners and opinions change, with the change of circumstances, institutions must advance also to keep pace with the times*.

-Thomas Jefferson

## Background

COVID-19 has been a focus of global concern since the beginning of 2020, and research literature, social media, and other information resources related to COVID-19 are being generated at high speed and in unprecedentedly large quantities [34]. In the face of an unknown virus, scientific research has the power, alongside medical treatment and health management, to guide us through unprecedented times.^1^ Moreover, scientific research paves the way for effective policy-making as well. Policy acts as a crucial tool and priority behaviour [28] for social public management. This fact partially explains the explosion of papers during the COVID-19 pandemic. In the face of a public health emergency, policies need to coordinate efforts to combat COVID-19; thus scientific research is included. Consequently, the dynamics between science and policy (SAP) can be understood using a coevolution model (CEM).

The concept of coevolution was originally proposed by Ehrlich and Raven [18]. Four policy-making models were proposed by Zwanenburg and Millstone [92], among which CEM is the only nonlinear model. The CEM reflects two-way feedback and continuous adjustment between SAP until a symbiotic equilibrium is reached under the influence of social and other factors, rather than simply a two-way influence. The CEM is regarded as the best model for effectively capturing the interactions between SAP-making [17, 22, 73]. Policies provide application-oriented research directions for science [13], accelerate the utilization of discoveries [57], and facilitate optimal resource allocation. The policy aids in constructing a denoising mechanism under the CEM to constantly screen for more appropriate scientific evidence to adopt. A new perspective on the relationship between SAP that differs from a more subjective model, the technocratic and decisionist models, has been shaped under CEM [53, 55]. Adjusting scientific evidence for policy-making under CEM is consistent with the goal of achieving evidence-based policy-making (EBPM). EBPM involves the design of policy based on evidence, which embeds scientific evidence throughout the process from policy formulation to evaluation to ensure that policies are scientific, effective, and reasonable [40, 64]. Thus, the dynamics between SAP under CEM meet the goal of EBPM effort [53] and provide a starting point to deeply explore EBPM [85].

One of the main challenges currently encountered during the construction of evidence-based policy is how to effectively transfer scientific evidence in policy-making [4, 32, 64]. The final quality of policy is determined based on the efficiency of elaborately refined scientific evidence operation in all processes constituting policy-making. Scientific evidence adopted in policy is invisible and unavailable to summarize at length to most people other than policy-maker themselves, which increases the difficulty in measuring the efficiency of scientific evidence utilization. Therefore, most of the current studies on the efficiency of scientific evidence operation dwell on the theoretical level [78, 87]. Major trends of scientific research and the corresponding policy changes in the early phase of COVID-19 were concluded, laying the basis for capturing the traces of scientific evidence in other policies without information source. It is also one of the innovative points of this study.

China was selected as a representative case for investigation for the following two reasons: First, China is among the first batch of countries identifying the COVID-19 epidemic. For instance, Weible et al. [77] reported that countries with early outbreaks, such as China and Italy, provided an opportunity for other countries to detect the pandemic and assess early policy responses. Second, China addresses the policy-making process standing on scientific research on COVID-19, but lacks relative research for mainland China. Professor Gao Fu, the Director-General of the Chinese Center for Disease Control and Prevention, claimed that the main objective of scientific research in the early period of the COVID-19 outbreak in China was to offer more reasonable reference and judgement for policy-making at the seventh academic conference of the academic divisions, Chinese Academy of Sciences. Yin et al. [85] recently published an article in *Science*, revealing the coevolution between SAP-making during the COVID-19 pandemic in 114 countries other than mainland China. Atkinson et al. [3] considered the dynamics of the United Kingdom policy response to the COVID-19 pandemic and explored how COVID-19 policy-making shares links with scientific research in the United Kingdom in order to capture real-time information. Therefore, the current study is complementary to CEM study on COVID-19. Third, this study provides a way to measure the dynamics between policy and scientific research when the policies lack references to the original scientific findings. Unlike other countries or regions, most policies in China do not publish reference sources and are not indexed in databases such as Overton, increasing the difficulty encountered in determining the association of Chinese policies with other information. To investigate the relationship between SAP without reference sources, dynamic trends in scientific research were analysed and identified via machine learning clustering analysis of scientific publications. Trends in relevant policies that correspond to changing trends in scientific research were then traced. This method is in contrast to the use of Altmetric data [33], analogy of numbers between publication and citation [73, 85], and field investigation of policy-making processes in departments involved in policy-making [41, 86] to analyse the policy–science evolutionary relationship. There are two other innovative points of this study.

The time scope of this research is limited to the early stage of COVID-19 in China. This was a period of high uncertainty. With increasing awareness about the virus, scientific research, medical defence and control, and policy-making all needed to continue making ongoing adjustments. With the deep insight and more focus on the virus in the later stage, the need to update scientific research and policy-making decreased gradually (please refer to Additional file 1: Table S1), and thus the interactions between SAP were less frequent and evident than those at the earlier stage. The white paper “Fighting Covid-19: China in Action” issued by the State Council of China reported that nationwide virus control was then being conducted on an ongoing basis from 29 April 2020. However, sporadic cases such as the epidemic outbreak in Beijing in May 2020 had been reported in mainland China. Consequently, the time scope of research cut-off was before 26 June 2020, roughly half a year after China’s official disclosure of COVID-19. Second, the earlier the epidemic is brought under control, the fewer losses society must suffer. The outcomes of prevention and control of the epidemic in the early phase played a more vital role in its overall development than in other stages, making this study informative for early prevention and control of emergencies.

The developmental trends of China’s COVID-19 research were analysed from the perspective of bibliometrics. Scientific research is a process of problem-solving and resolving disputes [21, 36]; therefore, trends of scientific research indirectly reflect whether a scientific consensus has been reached or whether a solution has been developed for a particular problem. Such connections can be revealed when one studies the length of time for which the particular topics remain popular. To clarify the dynamic trends of research, a total of 16 statistically valid time intervals were used. This study focused on the period from 30 December 2019^2^ (the initial announcement of the pandemic) to 26 June 2020, corresponding to a total of 18 intervals. Since the first COVID-related publication from China in PubMed was published on 24 January 2020, the total number of valid intervals is 16 (Additional file 1: Table S2). By calculating the variations in the co-occurrence of items over a range of time intervals, the research confirmed the items with a significant change and conducted a cluster analysis. It was found that in the later stage of the epidemic in China, the trend of research on “children and pregnant patients”, “common symptoms”, “nucleic acid test”, and “non-Chinese medicine” began to decline. In contrast, research on “aged patients”, “severe symptoms and asymptomatic infection”, “antibody assay”, and “Chinese medicine” began to rise. Mental health is a long-term “hot” issue in China’s COVID-19 research. The formulation of relevant COVID-19 policies in China is constantly evolving, and this is partially in response to these dynamic variations in COVID-19 research. In the early stage of the outbreak in China, the formulation of COVID-19 policies followed a rapidly progressing CEM. It also signifies China’s efforts to build EBPM during public health emergencies.

## Methodology

### Data collection

The research data for this study were derived from PubMed. The alternate names of COVID-19 provided by the Dimension database^3^ were used as search terms.^4^ The types of literature to be searched were limited to articles and reviews. As of 26 June 2020, the PubMed database had collected a total of 16 739 publications on COVID-19 research, among which 3708 papers were from China. The scheme used in this study adopted an expanding overlapping aggregation/overlapping time series approach [9, 39, 43]. A total of 16 statistically valid time intervals were used, following the examples provided by Petropoulos and Makridakis [60] and Roosa et al. [62]. Specifically, the time intervals were from 30 December 2019 to 28 January 2020, from 30 December 2019 to 7 February 2020, from 30 December 2019 to 17 February 2020, and so on, cumulatively increasing in 10-day increments until 26 June 2020. The specific intervals and corresponding dates are presented in detail in Additional file 1: Table S2. This strategy of collecting data in overlapping time series can reduce the influence of randomness on the variability of data in short periods and thus can present more stable trends than is possible with the use of continuous time series [9, 43]. Simultaneously, to avoid any bias introduced by the selection of the duration of the time interval, a robustness test (see details in Additional file 1: Appendix S3) was conducted. It was found that the variation significance values of co-occurrence keywords calculated under the 10-day interval scheme and under the 20-day interval scheme were highly correlated, with a correlation (*R*) value ranging from 0.77–0.93 (for six overlapping time intervals). These correlation ranges indicate that the variation significance of co-occurrence keywords can be effectively demonstrated by using different time interval schemes. In particular, the 10-day interval scheme is able not only to reveal more significant variations in the number of COVID-19 papers, but also to reasonably avoid the random fluctuations to which an excessively short interval would be susceptible.

### Data processing

All selected literature was imported into VOSviewer (version 1.6.16) in MEDLINE format for co-occurrence analysis. When setting the analysis conditions, the minimum number of occurrences of a keyword was set as 2, indicating that the keyword appeared in at least two documents. The data on the total link strength of co-occurrence items in different time intervals were extracted to calculate the differences in co-occurrence items. It not only recorded the occurrence frequency of a given item, but also reflected the link strengths of other items appearing at the same time as the given item [16].

The data on the total link strengths of corresponding items varied significantly because of the significant differences in the numbers of papers published during different intervals of different durations. According to the results exported from VOSviewer, the maximum total link strength in the first interval was 73, while that in the last interval was 39 814. To make the subsequent analysis more consistent, it was necessary to first normalize the total link strengths by transforming their values into percentages.^5^

### Identification of items with significant variations

Given that the number of co-occurring items varied in different intervals,^6^ data imputation was conducted to determine the missing percentages of the total link strengths of co-occurrence items to facilitate subsequent analysis. The missing data fell into the category of missing not at random; thus this study adopted minimum value imputation as its method [42]. Specifically, the minimum percentages of the total link strength of co-occurrence items in the 16 intervals were extracted separately. Then, data were randomly selected from the range constituted by the 16 minimum percentages for imputation with a 90% confidence interval. After imputation, a one-sample *t*-test was conducted on the percentage of the total link strength of each co-occurrence item in one interval relative to all prior intervals. This was done according to the following formula:

$$t \, = \overline{X} - \mu_{0} /{\text{SE}},$$

where $\overline{X}$ denotes the average percentage of the total link strength of the co-occurrence item in all prior intervals, *μ*_0_ denotes the percentage of the total link strength of the co-occurrence item in the current interval, and the standard error represents the error of the percentages of the total link strength of the co-occurrence item in all prior intervals.

According to the rules of the one-sample *t*-test,^7^ significance analysis could not be conducted on the data of the first and second intervals; thus ultimately 14 groups of *t*-values were obtained. Next, Student’s left-tailed *t*-distribution test was conducted on the *t*-values to calculate the significance of the data variations of co-occurrence items across different intervals. Any co-occurrence item having a *p*-value less than 0.05 was considered significantly changed at that time interval. To avoid any differences in the significance of data variations caused by random imputation, this study performed 10 random imputation iterations on the entire data set. The final imputation and *t*-test results adopted the average results of 10 random imputation iterations. According to *t*-test results, the 14 intervals (starting with the third interval), respectively, showed 25, 28, 37, 45, 78, 28, 31, 36, 40, 42, 49, 54, 51, and 56 co-occurrence items (162 in total after deducting redundancy) which showed significant variations in at least one interval.

### Classification of items

**Fig. 1:**
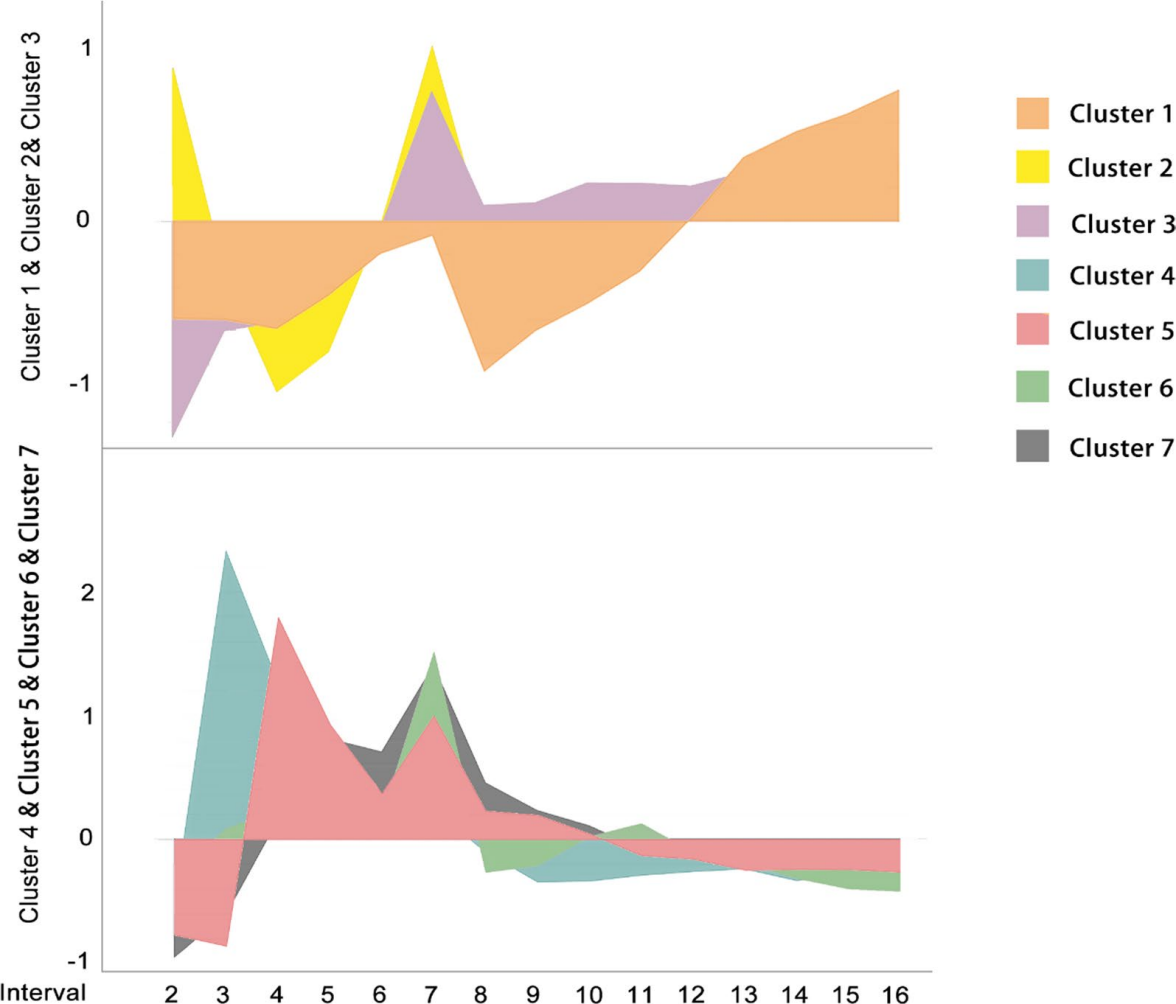
Heat variation trends of different clusters across different intervals

The interrelationships of the 162 co-occurrence items, which showed significant variations, were explored by classifying them through hierarchical clustering. In light of the significant differences among different items in their percentages of total link strength, *Z*-score transformation was first performed on the data for each item across the 15 intervals. Further, scikit-learn^8^ was used to analyse the transformed data to produce a dendrogram [59]. The dissimilarities among co-occurrence items were calculated according to average linkage and Euclidean distance metric parameters. According to the exported dendrogram (more details please refer to Additional file 2: Cluster mapR1), the 162 items were classified into seven major clusters based on their variation trends in research “heat”. Figure 1 shows the variations in research heat of all items in each cluster across different intervals, as measured by using *Z*-score. A high amplitude in Fig. 1 represents a steady increase in research focus rather than an instance of constant high research focus. These amplitude variations are referred to as “heat variations” in the remaining part of this paper. Among them, clusters 1–3 consisted of 80 words presenting a continuous increase in the later stage, while the heat variations of 85 words in clusters 4–7 gradually decreased in the later stage.

## Results

### Stage 1: analysis results of trend variations

Items that showed significant variations were grouped based on available classification, and five representative groups were selected to analyse their heat trend variations as follows:

### Patients: a shift from children and pregnant women to aged patients

**Fig. 2:**
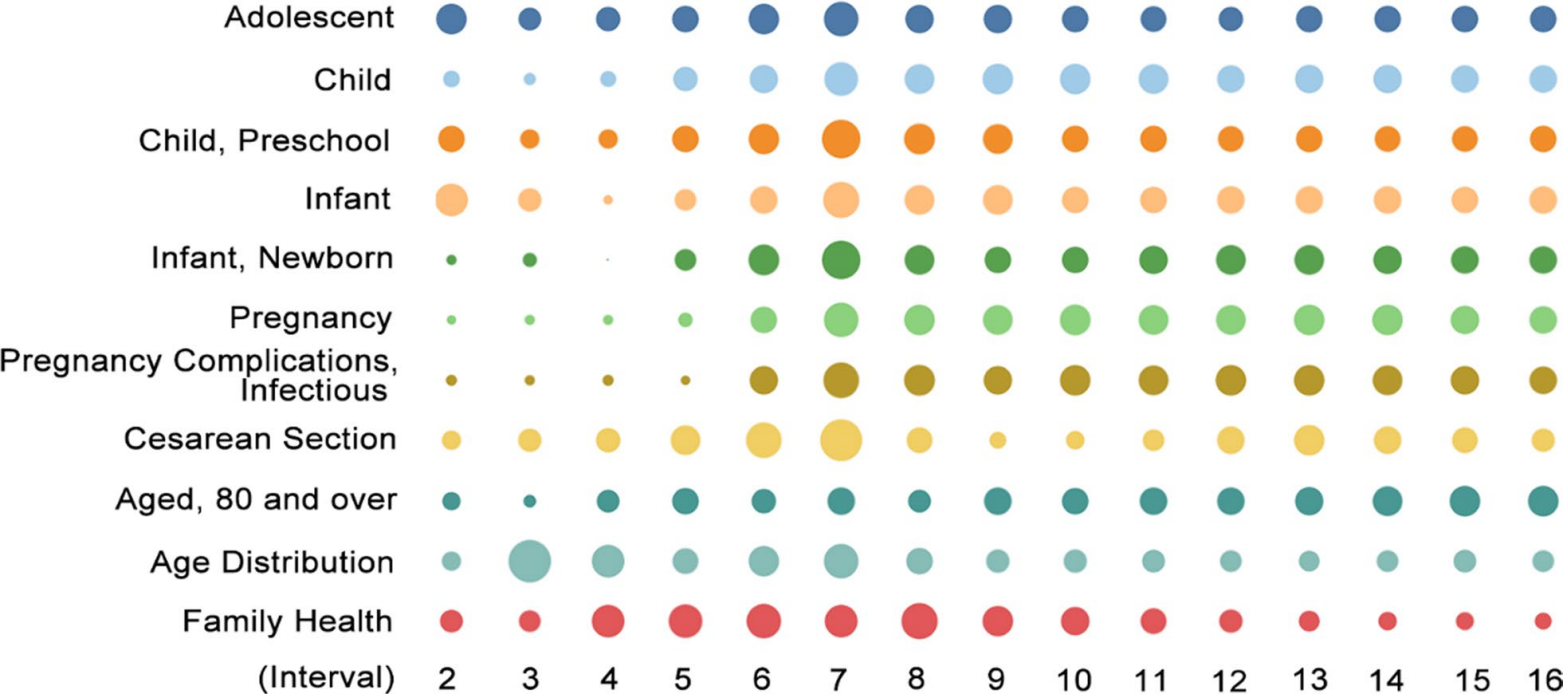
Heat variation trends of items related to children, pregnant women, and aged patients across different intervals

According to the data, the focus on child-related items such as “child”, “child preschool”, and “infant” peaked twice in the second and seventh intervals, while the terms “infant, newborn”, “pregnancy”, “pregnancy complications, infectious”, and “caesarean section” corresponding to pregnant women peaked only in the seventh cycle (Fig. 2). Children and pregnant patients present a special set of potential problems, such as a longer incubation period [79], with most recovering within 1–2 weeks after onset [38]. Pregnant women are susceptible to respiratory pathogens due to changes in immune mechanisms and physiological adaptations during pregnancy [47]. In the early stage, owing to the severity of the epidemic, the greatest concern was on the large number of patients, covering the special populations; however, during the middle stage of the epidemic, affecting the overall prevention and control of the epidemic, focus was on the patients belonging to remaining key populations—for example, the inability of some paediatric patients to describe the route of infection—and these presented difficulties to later prevention and screening [67].

The heat variations of the items related to the above-mentioned patient types declined in the later stages as the epidemic eased,^9^ while that related to aged patients increased. The heat variations of the item “aged 80 and over” gradually increased. Compared to younger people, the aged population experienced more severe symptoms [29] and higher mortality rates [48, 80] due to underlying or previous diseases. Considering that most of the existing patients in the later stage of the epidemic in China are severe cases and also that aged patients recover more slowly after infection [72], the aged patients may become the main patient population in late surviving cases.

### Clinical characteristics: a shift from common symptoms to severe symptoms and asymptomatic infection

**Fig. 3:**
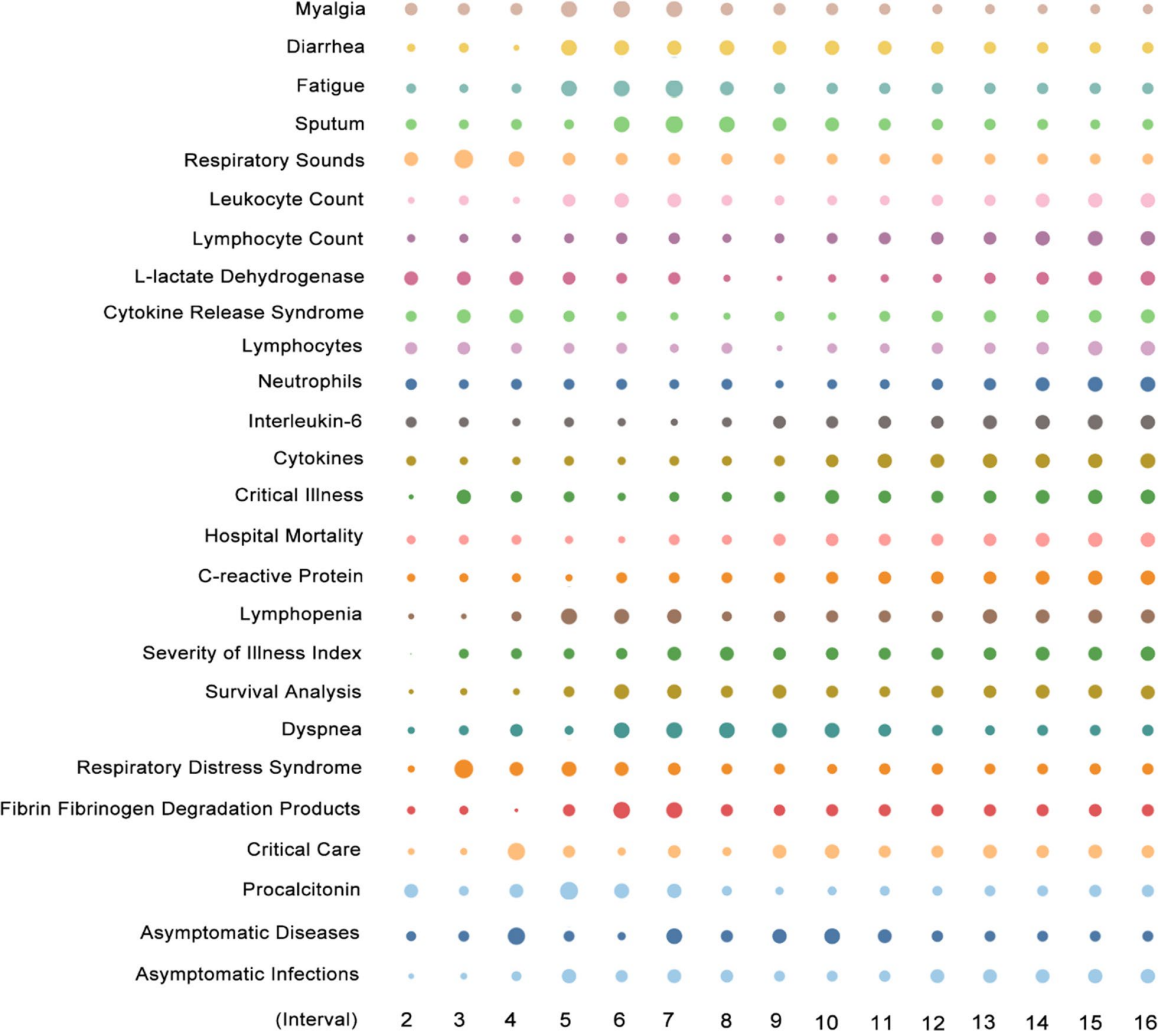
Heat variation trends of items related to symptoms across different intervals

The focus on the most severe illness-related items such as “L-lactate dehydrogenase”, “cytokine release syndrome”, “interleukin-6”, “critical illness”, “hospital mortality”, “C-reactive protein”, and so on increased in the eighth or ninth interval (Fig. 3). The items “leukocyte count”, “lymphocyte count”, “lymphocytes”, and “neutrophils” (neutrophil–lymphocyte ratio) are not specific to severe disease, but clinical characteristics of severe cases present in a different manner than common cases. These severe disease-related items have also been found to be the most relevant to the studies in later stages [51, 61, 83]. The symptoms or indicators of common cases, such as “myalgia”, “diarrhoea”, “fatigue”, “sputum”, and “respiratory sounds”, began to decrease after the third to seventh intervals, respectively, in contrast to the heat variation of the terms relevant to severe cases. However, the clinical characteristics of common cases reached a consensus in the early stages, and the indicators of severe symptoms are in constant turnover due to the complexity of severe disease treatment [46]. Treatment for critical patients still has much room for improvement, as the mortality rate remains high, and thus most studies are constantly updating the critically ill indicators [31, 37, 90].

Notably, asymptomatic infections have been continuously increasing after the sixth and 11th interval in heat variation. Although trends of asymptomatic diseases and asymptomatic infections present in a distinct way, a complementary state exists between their heat variations. Asymptomatic disease-related research is a latecomer. The initial prevention and control of COVID-19 focused on symptomatic patients. However, with the continuation in further studies, most patients were found to suffer from mild symptoms or were asymptomatic [91]. Asymptomatic patients create new pressure for outbreak prevention and control, and whether they are infectious or not remains controversial [23, 25]

### Virus testing: a shift from nucleic acid tests to a combination of nucleic acid tests and antibody assays

**Fig. 4:**
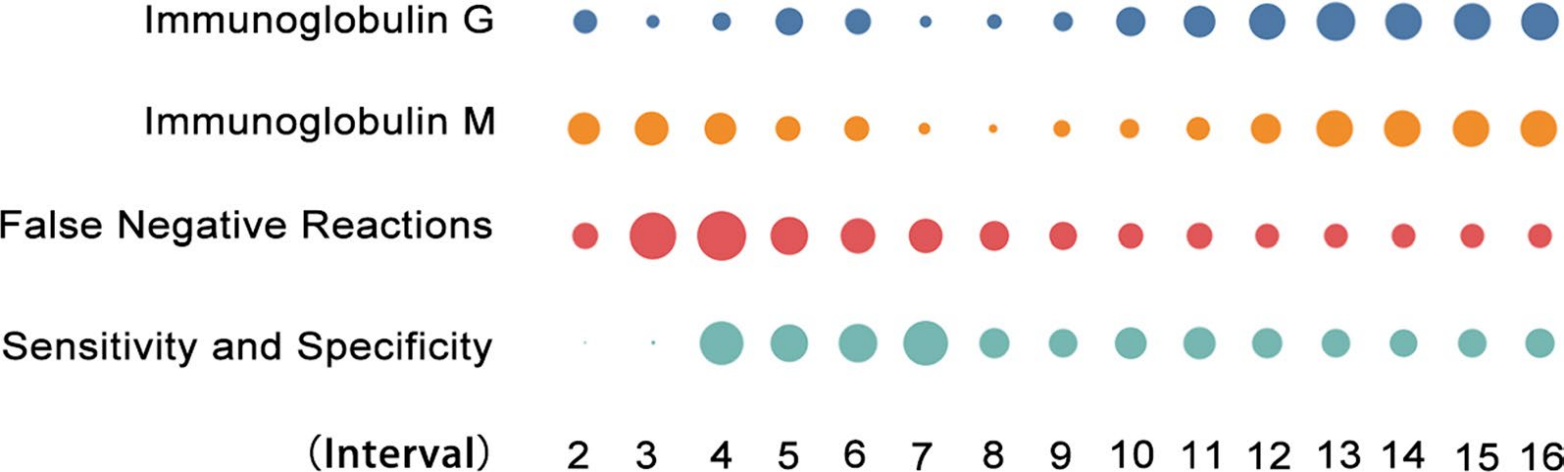
Heat variation trends of items related to virus testing across different intervals

Nucleic acid test results came under question by many academics in the early stage, due to the issue of false negatives [82, 84] and sensitivity [71]. Therefore, the terms “false negative reactions” and “sensitivity and specificity” increased steeply after the second and third intervals. The problem of false negatives was gradually resolved with the improvement of technology; however, the academic community still emphasized the sensitivity and specificity of the assay [10], with the result that the heat variation of “sensitivity and specificity” lasted longer than that of “false negative reactions”. In the late stage of the epidemic, when the numbers of suspected and confirmed cases in China declined, antibody assays helped to assure a safe reopening of the economy. At the same time, with the emergence of asymptomatic and imported cases, antibody testing has become an important approach to map previous infections, and therefore, later research on virus testing has shifted to antibody testing. The increase in the vital indicators of “immunoglobulin G” and “immunoglobulin M” continued after the ninth interval (Fig. 4).

### Drug research: a shift from non-Chinese medicine to Chinese medicine

As the demand for drugs has gradually decreased because of epidemic mitigation in China, most of the items related to drugs show the trend of dropping in the late stage of research, such as “drug therapy, combination”, and the existing drugs “chloroquine”, “indoles”, “lopinavir”, and “ritonavir”. Moreover, researchers’ perceptions of the effects of some drugs changed based on improved research. Early drug adoption relied on severe acute respiratory syndrome (SARS) and Middle East respiratory syndrome (MERS) treatments, such as ritonavir [58], but later, it was found that several medicines such as chloroquine or hydroxychloroquine were associated with huge side effects [6].

**Fig. 5:**
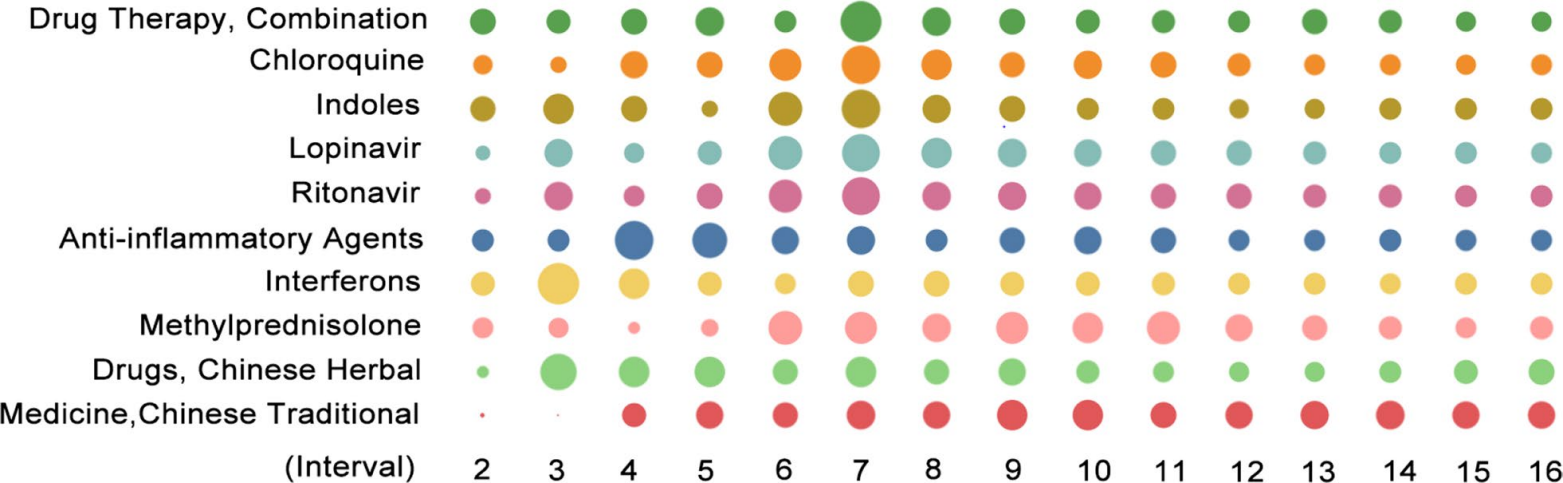
Heat variation trends of items related to drug research across different intervals

Of note, the heat variation of Chinese medicine-related items continues unabated. The heat of “drugs, Chinese herbal” started to increase after the 13th interval, although it declined in the mid-term, and the focus on “medicine, Chinese traditional” increased progressively after the fourth interval and then stabilized. During COVID-19 treatment, a variety of Chinese herbal medicines not only possess obvious efficacy when combined with Western medicine, but also can alleviate the side effects brought by Western medicine. One example is the use of Qingfei Paidu decoction combined with Western medicines such as lopinavir and interferon α2b injection [89]. Lianhua Qingwen capsules exhibit a higher safety profile than the antiviral oseltamivir in the treatment of critical and severe cases of influenza A virus subtype H1N1 in children [52] (Fig. 5).

### Long-term attention to mental health

**Fig. 6:**
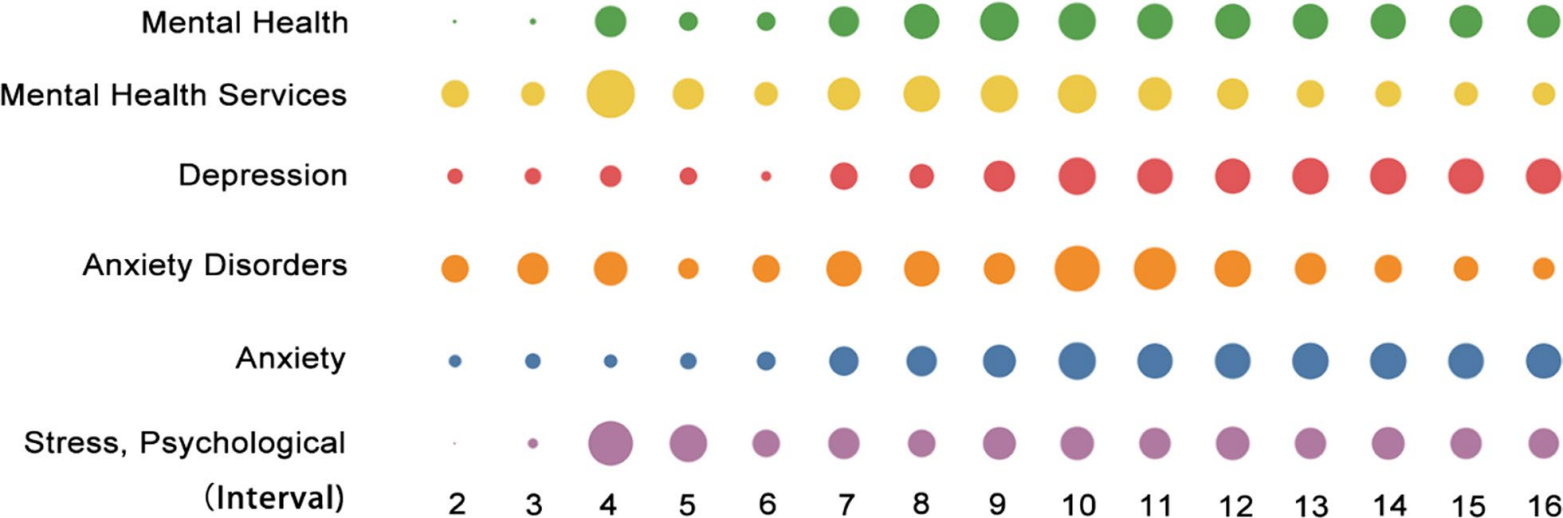
Heat variation trends of items related to mental health across different intervals

The public’s mental health suffered from COVID-19 and may take a longer period to recover compared to physical health. China also began to deploy mental health surveys in January in order to determine the impact of COVID-19 on public mental health [88]. Figure 6 demonstrates that increase in the heat of different research topics related to mental health appeared successively over time; thus overall, mental health maintained a long-term research focus. Research on mental health in China paid close attention to medical staff and people at the epicentre [49] in the early stage of the pandemic, and the attention shifted to the public at large in the middle and late stages [69].

### Stage 2: analysis results of policy-making

According to data from China’s National Health Commission, 148 policies related to COVID-19 were issued between 30 December 2019 and 26 June 2020.^10^ This study compared the onset of the period of maximum heat in each cluster and the issuance date of the corresponding national policy/guidance programme for each of the five trend changes (Table 1. See the list of policies presented in Additional file 1: Appendix S5). By calculating the differences between the two, an average interval of 8.36 days was found between the issuance of a policy and the maximum heat period of research on related topics. The increase in interest for various clusters followed the release dates of relevant policies and guidelines, which may reflect that the policy plays a certain leading role on related research topics. For example, Diagnosis and Treatment Protocol of COVID-19 (Trial Version 7) released on 4 March 2020 specifies clinical early-warning indicators of severe and critical cases for the first time; these indicators include cytokines, interleukin 6, and C-reactive protein. The significance of items relative to these three indicators began rising extensively after 18 March. Moreover, some particular policy outcomes impacted the scientific research, as reported by some literature studies. For example, when referring to the Prevention and Control Protocol of COVID-19 (Trial Version 6), Ge et al. [26] added that aerosol transmission is a potential transmission route. Liu et al. [50] conducted an experimental study of patient inclusion criteria based on the Diagnosis and Treatment Protocol of COVID-19 (Trial Version 5).

**Table 1:** Stages of rising heat for various clusters in China’s COVID-19 research and release dates of relevant policies/guidelines

Cluster	Waves	Release dates of relevant policies or guidelines (a)	Stages of rising heat for clusters of research topics (b)	Interval between the first date of (a) and that of (b)
Children and pregnant patients	First	1.28–4.09	1.28–2.07	0
	Second	5.08–6.01	2.27–3.28	
Aged patients	First	1.29–2.26	1.28–2.07	1
	Second	4.08–5.29	2.17–6.26	
Severe symptoms	First	1.28–4.11	2.07–3.08	9.5
	Second	5.08–6.08	5.17–6.16	
Asymptomatic infection	First	2.04–2.21	2.07–2.27	12
	Second	4.08–6.08	3.18–3.28	
Antibody test	–	4.05–5.08	4.08–5.27	3
Chinese medicine	First	1.26–3.04	2.07–3.08	12
	Second	5.14–5.22		
Mental health	First	1.27–5.08	2.17–2.27	21
	Second		3.18–4.27	

**Note:** The number of days in the last column between the first date of “release dates of relevant policies or guidelines” and the first date of “stages of rising heat for clusters of research topics” is taken from the average of the date interval between the two waves of each cluster. Among them, the period of rising heat of terms related to children, pregnant women, and Chinese medicine is included in the period of concentrated promulgation of related policies/programmes; thus, only the time difference between the period of concentrated promulgation of the first wave of related policies/programmes and the period of concentrated heat of related terms is counted

The progress of scientific research is also of vital significance for policy-making [28, 86] and promotes the adaptive adjustment of policies [81]. In the first 2 months after announcing the pandemic situation, the Chinese government adjusted its diagnosis and treatment protocol seven times and revised its protocol for prevention and control six times. The data indicate that the increased focus on some items preceded the release time of relevant policies or guidelines (Table 2), and some of the studies examined herein explicitly suggested that their research results should be used to guide policy. For example, Gao et al. [24] proposed in papers published on 4 February and 19 February, respectively, that chloroquine is an effective treatment for COVID-19, and suggested that chloroquine should be included in the diagnosis and treatment protocol.

**Table 2:** Stages of rising heat for different items in China’s COVID-19 research and release dates of relevant policies and guidelines

Item	Stage of rising heat	Relevant policies or guidelines in the early stage		Relevant policies or guidelines in the late stage	
		Name	Release date	Name	Release date
Chloroquine	2.17–2.27	Diagnosis and Treatment Protocol of COVID-19 (Trial Version 6)	2.19	Notice on Adjusting the Usage and Dosage of Chloroquine Phosphate in Treating COVID-19 on a Trial Basis	2.28
Mask	2.17–3.18	Notice on Issuing the Technical Guidelines on the Selection and Use of Masks Used for Prevention and Control of COVID-19 in Different Populations	2.05	Notice on Issuing the Guidelines on the Scientific Wearing of Masks	3.18
Infectious disease transmission, patient-to-professional	3.18–4.17	Notice on Further Strengthening Protection of Medical Staff during COVID-19 Prevention and Control	2.19	Notice on Further Strengthening Infection Prevention and Control at Medical Institutions during COVID-19	3.13
Telemedicine	2.17–2.27	Notice on Effectively Conducting Internet-Based Diagnosis, Treatment, and Consultation Service Work during COVID-19 Prevention and Control	2.07	Notice on Conducting Online Services to Further Strengthen COVID-19 Prevention and Control Work in Hubei Province	2.26
Patient discharge	2.27–3.08	Diagnosis and Treatment Protocol of COVID-19 (Trial Version 6)	2.19	Diagnosis and Treatment Protocol of COVID-19 (Trial Version 7)	3.04

The Diagnosis and Treatment Protocol of COVID-19 (Trial Versions 6 and 7), released on 19 February and 4 March, respectively, both included chloroquine as a recommended drug. Later, with the further enrichment of clinical trial data on chloroquine, the “Notice on Adjusting the Usage and Dosage of Chloroquine Phosphate in Treating COVID-19 on a Trial Basis”, published on 28 February 2020 further adjusted the usage and dosage recommendations for chloroquine. Moreover, “masks”, “infectious disease transmission, patient-to-professional”, “patient discharge”, and “telemedicine” also showed similar time differences between the stage of rising heat of an item and the release of relevant policies or guidelines (see Table 2). Thus, the research foci identified in this study may affect the formulation of COVID-19 policies and guidelines in China.

## Discussion

Notably, this study did not trace scientific research evolution supported by policy attributed to the lack of solid references presenting connection between policies and research output. However, in this study, it was observed that the trends of scientific research and related policy changes were closely intertwined in the early stage of COVID-19. This result is based on the calculation of the coincident periods between heat variation in the main themes of scientific research and the centralized enactment of relevant policies. A similar dynamic trend is a manifestation of two-way feedback and adjustment which was emphasized by the CEM. Furthermore, the available evidence indicates that early COVID-19 scientific research and related policies influenced each other. Consequently, the CEM of policy-making was reflected in the early stage of the outbreak in China, consistent with the policy documents published by government agencies and think tanks from 114 countries as presented by Yin et al. [85]. This result is opposed to the opinion proposed by Khazragui and Hudson [44] and Haunschild and Bornmann [33], that only a single piece of research has a decisive influence on policy. Policy-making and scientific research are both gradual processes. Scientific research is the practice of resolving scientific disputes [14] and aims to approach the truth [2]. Given that policies are usually a step behind technological development, it is necessary for them to be constantly updated [54] or to be given adaptive adjustments in response to emerging issues [81]. Policy-makers rely on the participation of other parties concerned with the dynamic adjustment of policies, and scientists within the same sociotechnological circles need to maintain continuous interactions with policies [17, 70]. Importantly, the mission of modern science is not only to create new knowledge, but also to use existing and new scientific knowledge to solve societal problems [63]; thus the policy-making process concerning or depending on science and technology needs to be guided by scientific knowledge [27, 77]. The Global Preparedness Monitoring Board calls for responsible leaders to act decisively based on science, evidence and best practices, and the interest of the people. Zhang et al. [89] also argued that the findings of academia pertaining to public health emergencies may offer a key reference for health policy-making. The Chinese government addressed this during the formulation and revision of the diagnosis and treatment and other protocol for COVID-19, wherein the available medical research evidence was fully considered. Moreover, they scientifically and prudently maximized consensus-building in the press conference of the Joint Prevention and Control Mechanism of the State Council.^11^ It is additional proof of the existence of CEM in Chinese COVID-19 policy formulation.

The coevolution between SAP in the early phase of the COVID-19 pandemic in China also shows individual characteristics that distinguish it from those of other countries. This can be discussed from the following two perspectives:

First, although most of China’s COVID-19 policies are promulgated by the government, the role played by scientific experts in the formulation of some policies is elevated from that of adviser to decisionist. All 148 new Chinese policies related to COVID-19 were promulgated and coordinated by government agencies. This result is in sharp contrast to the conclusion by Yin et al. [85], who reported a low likelihood of the national government citing scientific papers in formulating COVID-19-related policy. About 36.49% of all policies were enacted by the Joint Prevention and Control Mechanism of the State Council in Response to the Novel Coronavirus Pneumonia. The mechanism was launched by the State Council on 20 January 2020 to respond to the severe and specific infectious pneumonia epidemic, with the establishment of a Scientific Research Group, including teams belonging to Nanshan Zhong, Lanjuan Li, and Chen Wang.^12^ In addition to the medical treatment on the front line, these teams incorporated some effective clinical experience into the treatment protocol, which provided scientific and reasonable judgements and recommendations for the prevention and control of the pandemic, and further revised and improved relevant prevention and control measures. Of note, the Scientific Research Group was able to enact certain medical policies without the involvement of politicians. For example, on 25 February 2020, the Scientific Research Group of the Joint Prevention and Control Mechanism of the State Council in Response to the Novel Coronavirus Pneumonia issued a “Notice on Regulating Medical Institutions to Conduct Clinical Studies on the Drug Treatment of Novel Coronavirus Pneumonia”. Therefore, in China, the COVID-19 policy-making approach adopted an “advisers advise and decide” model in the early period, which is different from the “advisers advise and ministers decide” model used in the COVID-19 policy-making in the United Kingdom [3]. Moreover, the “decisionist model” of China’s industrial policy-making was discussed by Chen et al. [11]. The “advisers advise and decide” model may be the fruit of the establishment of an advisory system for the formulation of major science and technology policies in recent years [53]. Although most Chinese policies are initiated or coordinated by the government [68], China has attempted to adjust its policy-setting agenda to be more scientific [75]. Two examples are presented as follows: the establishment of the National Science and Technology Decision-making Advisory Committee in 2017 [19], and an attempt to develop the National Outlines for Medium and Long-term Planning for Scientific and Technological Development (2006–2020, 2021–2035) with the joint participation of the Chinese Academy of Sciences, the Chinese Academy of Engineering, and the Chinese Academy of Social Sciences in the “strategy consultation” mechanism [74].

Second, in China, early policy-making on COVID-19 gave more importance to prior clinical practice, unlike other countries that relied more on statistical modelling. The results of clinical or prevention practice became the main reference for the development of the Diagnosis and Treatment Protocol and Prevention and Control Protocol of COVID-19 in China. The development of COVID-19-related policy in China embraced a model of policy-making while practicing. In other words, whether decisions based on scientific research should be included in policy documents is based on the results of preliminary clinical trials, and revisions are kept for later if any new results are found. The concept that policy-making goes hand in hand with practice is distinct from the traditional notion of some countries that rely on modelling in their policy-making process, for example, during the 2009 swine flu epidemic in the United Kingdom [5]. Modelling remains an important reference for policy formulation in Europe and the United States during the COVID-19 outbreak [12]. In contrast, clinical practice provides more accurate reference information than modelling, and takes into account differences in effects brought by various objects or settings. Modelling provides the reference information at a relatively fast rate and eliminates the need for and risk of conducting clinical investigations in patients under conditions of considerable uncertainty in the early period [35, 65]. Boden and McKendrick [7] considered modelling the most ethical method. Modelling results are also included in consideration of the initial phase after weighing the pros and cons between practice and modelling, such as in case of hydroxychloroquine. About 100 drugs were selected for in vivo experiments on the activity of the novel coronavirus via computer simulation screening and so on. Based on multiple rounds of screening, the Scientific Research Group concentrated on a few drugs such as hydroxychloroquine.^13^ Hydroxychloroquine was recommended in the Diagnosis and Treatment Protocol of COVID-19 (Trial Version 6) according to the results of initial clinical trials. The clinical trial results for more than 100 patients were accumulated before listing the drug in the treatment protocol.^14^ However, the dose, drug regimens, and target patients of hydroxychloroquine standardized in the Diagnosis and Treatment Protocol of COVID-19 (Trial Version 7) were further tested based on additional clinical studies, which showed that overdose of chloroquine may cause damage to the heart and retina. Consequently, in the earliest stage, the candidate methods for treatment and prevention depend on the model results; nonetheless, the formal enrolment in the policy is principally judged by the practical results.

Excluding the difference from other countries, the coevolution of SAP under emergencies also presents some features apart from other domains: science tends to exert a rapid and direct influence on policy formulation in the early period of public health emergencies. The model of policy-making under peacetime is no longer applicable in times of war, such as in the COVID-19 scenario [3, 77]. The high frequency of COVID-19-related policy enactment in the early stages of the pandemic in China exhibited a rapid coevolution between SAP, demonstrating that the response to the emergency needs to run at a faster pace than pandemic development [3]. Importantly, this is different from its progress in the formulation of climate change policies, where it has proven to be difficult to reach a consensus between scientists and government officials even after much discussion [41, 76]. Edmondson [17] reported that the interactions between SAP with respect to the CEM are also affected by external factors such as catastrophic events. Considering the urgency and unknown nature of the epidemic, rapid response is essential to plan for and mitigate further impact [56]. Furthermore, the process of science–policy interaction during any public health emergency may be simplified, with SAP tending to form a direct relationship with each other. Van Zwanenberg and Millstone [92] addressed the concept of coevolution of SAP and mentioned that the interaction between SAP might be influenced by cultural, political, and other contextual factors. Gormley [27] argued that policy could be formed after public opinion on scientific issues was shaped by media coverage. The process of SAP interaction may be slowed with the increase in the influencing factors that need to be considered. In the early period of COVID-19, the government mainly focused on exploring and controlling the pandemic; as a result, the research, prevention, and control under important scientific guidance became the priority. Other themes such as economic recovery and social functioning began to be on the agenda of policy-making after the pandemic was under control. Notably, a difference has been reflected in the COVID-19-related policy between the early period and late period (Additional file 1: Appendix S5). It also proves that scientific information has been regarded as principal evidence to be considered in the early-period policy-making of public health emergencies, even science, and technology-based emergencies. The influence that science information brought to policy consultation can be possibly transferred from being direct and fast in the early period to conditional and uncertain in the later stage, as other factors such as culture, economy, and politics also start playing their role. As a result, the role played by science is destined to diminish to a supportive position.

Some controversial aspects of policy advisory science can also be observed. For instance, Weible et al. [77] mentioned that scientific research contributed to inform and legitimize decision-making, which could be used to obscure the government’s and policy-makers’ responsibility for policy responses and outcomes. Furthermore, Durnová [15] argued that emotions are a crucial part of policy-making. However, the role of emotions and their impact on legitimizing decisions and achieving desired outcomes are likely to be overlooked if excessive attention is focused on the role of scientific research.

## Strengths and limitations

This study presents a manifestation of the CEM for SAP, as well as an effective approach to measure the interaction between SAP, while policies simultaneously lack references to the original scientific findings. It not only favours the construction of unbiased approaches to extract scientific evidence for framing policies for future, but also may be beneficial to bridge the gap that exists between SAP, which is discussed based on extensive health and public health literature [8]. This research also serves as a complementary case to the dynamic relationship between SAP at the global scale during the COVID-19 outbreak. However, the other link for the mutual interaction between the two can be further explored by combing scientific paper citation information or interviewing experts and by other methods. Multiple types of scientific information are available for reference during policy formulation. The influence of scientific research on policy-making is not limited to scientific papers, but includes the interactions between scientists and policy-makers and clinical trials [66, 86]. For example, clinical trial results also act as reference for important scientific information, which may also be cited before they are published. This may also be a reason why Chinese policies do not offer scientific reference. Scientific information other than scientific papers needs to be obtained in the future. Moreover, in this study, it was also noticed that policy plays a certain role in leading research questions in COVID-19, and the examples are presented in part 3.2. How and to what extent policies prompt the scientific research questions could be a direction for our future research. The timescale of this study was about 6 months; future studies may find it profitable to use a longer timescale. A base time interval of 10 days was adopted to investigate variations in research trends, but there may be other better division schemes.

## Conclusions

Considering that scientific research is a process of solving problems and resolving disputes, the following findings can be drawn from this study. First, a similar dynamic trend was reflected between scientific research and related policy in early period of the COVID-19 outbreak in China, wherein an average interval of 8.36 days was observed between policy disclosure and the intensive publication period of related research. Second, issues such as aged patients, asymptomatic infections, critical care, and antibody testing may be of concern in the later stages of prevention and control. Mental health has remained under-researched and thus a significant challenge in China’s fight against COVID-19, and the sensitivity and specificity of nucleic acid tests still require improvement. Third, the application of Chinese medicine in the treatment of COVID-19 is gaining more recognition in China, while essential involvement of herbal medicine in the treatment of COVID-19 has been further improved.

In their review on the existing global COVID-19 literature, Haghani et al. [30] discovered that most studies have focused on drug safety, where clinical characteristics, treatment, mental health, and nucleic acid and antibody test research are particularly prominent among global studies [1]. This reflects the fact that these issues tend to be common problems faced globally that prompt the strengthening of international collaboration [45], resulting in an unprecedented intensity of international collaborative research during COVID-19.^15^ However, the specific circumstances of the epidemic vary from country to country, and based on this, each country has developed its own countermeasures, such as traditional Chinese medicine. Our study collected research trends of COVID-19 research in the United States which did not include Chinese medicine. Differences in pharmacology between Chinese and Western medical systems cannot be avoided; thus, studies on Chinese herbal medicine and COVID-19 appear more frequently in Chinese journals than in English journals [20].

## Supplementary Information

**Additional file 1: Appendix S1.** Number of issued policies and scientific publications on COVID-19 from 2020 January to 2020 December. **Appendix S2.** Time series in different time intervals. **Appendix S3.** Robustness tests. **Appendix S4.** Heatmap of items with significant variation. **Appendix S5.** List of policies.

**Additional file 2:** Heatmap of items with significant variation.

**Additional file 3:** Results output from VOSviewer (version 1.6.16).

## Data Availability

All data generated or analysed during this study are included in this published article, its Additional file 1 and 3.

## Abbreviations

CEM: Coevolution model
EBPM: Evidence-based policy-making
MERS: Middle East respiratory syndrome
SAP: Science and policy
SARS: Severe acute respiratory syndrome

## Footnotes

1 https://www.forbes.com/sites/startswithabang/2020/04/07/the-3-ways-science-will-get-us-through-the-covid-19-pandemic/#39ee6ca52fc3.

2 http://www.chinadaily.com.cn/a/202004/06/WS5e8b2f5aa31012821728496b_2.html.

3 https://covid-19.dimensions.ai/.

4 (("2019-nCoV"[All Fields] OR "COVID-19"[All Fields] OR "SARS-CoV-2"[All Fields] OR "hcov"[All Fields] OR "NCOVID-19"[All Fields] OR "severe acute respiratory syndrome coronavirus 2"[All Fields]) AND ("CHINA"[Affiliation])).

5 The percentage of the total link strength of a given co-occurrence item equals the total link strength of the given co-occurrence item divided by the sum of the total link strengths of all co-occurrence items.

6 The numbers of co-occurrence items in each of the 16 intervals were 22, 72, 109, 168, 254, 319, 396, 534, 675, 794, 913, 1043, 1162, 1265, 1347, and 1424.

7 A one-sample *t*-test compares target data and test data. The first step is to determine whether the data for a particular item in a particular interval differ from the data for that same item in the previous interval. If so, the data in that interval are adopted as target data, while the data in the previous interval are adopted as test data. Data from at least two previous intervals are required for comparison with the target data. For this reason, no *t*-test was performed on the second or third intervals. (Recall that the first interval was already discarded for other reasons.) After deleting these two intervals, 14 groups of *t*-values were ultimately obtained.

8 Scikit-learn is a Python-based machine learning library.

9 According to Worldometer, the number of confirmed cases of COVID-19 in China declined rapidly after mid-February 2020, in particular after March 6, when the number of daily new cases dropped below 100; thus the discussion about fever declined at a later stage.

10 These data are available as of 1 April 2021.

11 Referring to the press conference of the Joint Prevention and Control Mechanism of the State Council held on 5 February 2020.

12 Referring to the press conference of the Joint Prevention and Control Mechanism of the State Council held on 7 February 2020.

13 Referring to the press conference of the Joint Prevention and Control Mechanism of the State Council held on 15 February 2020.

14 Referring to the press conference of the Joint Prevention and Control Mechanism of the State Council held on 15 February 2020.

15 https://www.nytimes.com/2020/04/01/world/europe/coronavirus-science-research-cooperation.html
