# Supplementary material for: Coevolution of COVID-19 research and China’s policies
Source: Health Res Policy Syst. 2021 Sep 6;19:121. doi: 10.1186/s12961-021-00770-6 (PMC8419657; doi:10.1186/s12961-021-00770-6)
Supplement: Supplementary file 1 — Additional file 1: Appendix S1. Number of issued policies and scientific publications on COVID-19 from 2020 January to 2020 December. Appendix S2. Time series in different time intervals. Appendix S3. Robustness tests. Appendix S4. Heatmap of items with significant variation. Appendix S5. List of policies. [file 12961_2021_770_MOESM1_ESM.docx]

Additional file 1

**Appendix S1****.Number of issued policies and scientific publications on COVID-19 from 2020 January to 2020 December**

Table S1: Number of issued policies and scientific publications on COVID-19 from 2020 January to 2020 December

| **Time** | **Number of issued policies** | **Number of scientific publications** |
| --- | --- | --- |
| 2020 January | 29 | 12 |
| 2020 February | 64 | 219 |
| 2020 March | 22 | 543 |
| 2020 April | 17 | 1087 |
| 2020 May | 14 | 1059 |
| 2020 June | 3 | 788 |
| 2020 July | 15 | 592 |
| 2020 August | 5 | 588 |
| 2020 September | 5 | 562 |
| 2020 October | 0 | 586 |
| 2020 November | 2 | 537 |
| 2020 December | 0 | 565 |

Policy Source: China's National Health Commission

Publication Source: PubMed

Note: There were no policies issued or publications published on COVID-19 in 2019 December.

**Appendix S2. Time series in different time intervals**

Table S2: Time series in different time intervals

| **Time Series** | **Intervals** |
| --- | --- |
| 2019/12/30 – 2020/01/28 | 1 |
| 2019/12/30 – 2020/02/07 | 2 |
| 2019/12/30 – 2020/02/17 | 3 |
| 2019/12/30 – 2020/02/27 | 4 |
| 2019/12/30 – 2020/03/08 | 5 |
| 2019/12/30 – 2020/03/18 | 6 |
| 2019/12/30 – 2020/03/28 | 7 |
| 2019/12/30 – 2020/04/07 | 8 |
| 2019/12/30 – 2020/04/17 | 9 |
| 2019/12/30 – 2020/04/27 | 10 |
| 2019/12/30 – 2020/05/07 | 11 |
| 2019/12/30 – 2020/05/17 | 12 |
| 2019/12/30 – 2020/05/27 | 13 |
| 2019/12/30 – 2020/06/06 | 14 |
| 2019/12/30 – 2020/06/16 | 15 |
| 2019/12/30 – 2020/06/26 | 16 |

**Appendix S3. Robustness tests**

This study verified the variation significance of the co-occurrence of keywords under the 20-day interval scheme, to avoid the deliberateness of selecting 10 d as one time interval. When adopting 20 d as one time interval, this study had a total of eight valid time intervals within the study period, from December 30, 2019 to June 26, 2020. According to Footnote 8 in the main text, a one-sample t-test requires at least three samples. As a result, this study obtained six periods of t-test results (time-series: from December 30, 2019 to March 18, 2020, from December 30, 2019 to April 7, 2020, from December 30, 2019 to April 27, 2020, from December 30, 2019 to May 17, 2020, from December 30, 2019 to June 06, 2020, and from December 30, 2019 to June 26, 2020). Next, this study analyzed the Pearson correlation (R) between the six periods of t-test results under the 20-day interval scheme with the t-test results over the same six intervals under the 10-day interval scheme. This study found that they were highly correlated. The correlations between the t-test results under the 20-day interval scheme and those under the 10-day interval scheme over these six intervals were 0.77, 0.80, 0.87, 0.86, 0.91, and 0.93 respectively (as shown in Supplementary Figure 1). Thus, the variation significance of the co-occurrence of keywords can be effectively reflected using different interval division schemes, with only slight differences. However, the 10-day interval scheme obtained 162 items with significant variations. In comparison, the 20-day interval scheme only acquired 78 (72 within the 162 items), so adopting 10 d as one time interval can reveal richer and more detailed variations in items.


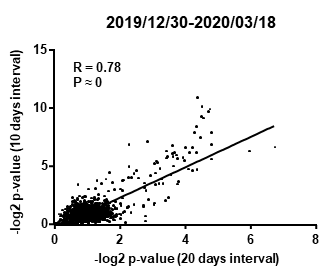

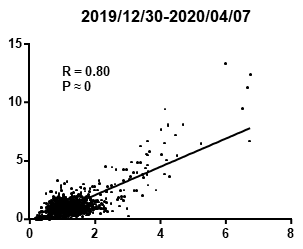


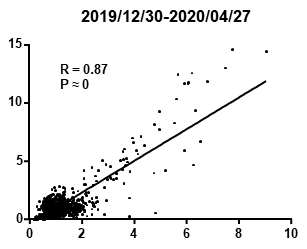

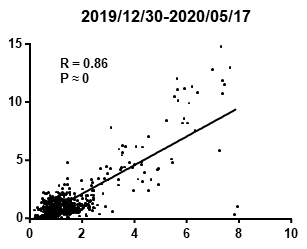


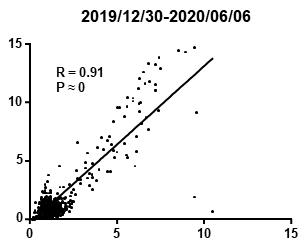

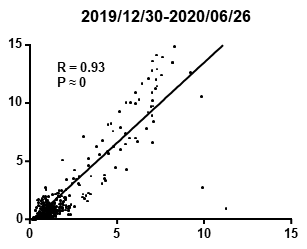


Figure S1: Keywords p-value correlation of 20 days interval

**Appendix S4. Heatmap of items with significant variation**

This study classified the items into seven major clusters based on their heat trend variations, and used a tree diagram of three to five levels. This classification method has been extensively used in studies by Tang and Hu (2013), Lee (2019), Cheng (2018), and Grembergen (2016), among others. Original image with high clarification please refer to “Cluster map.pdf”.


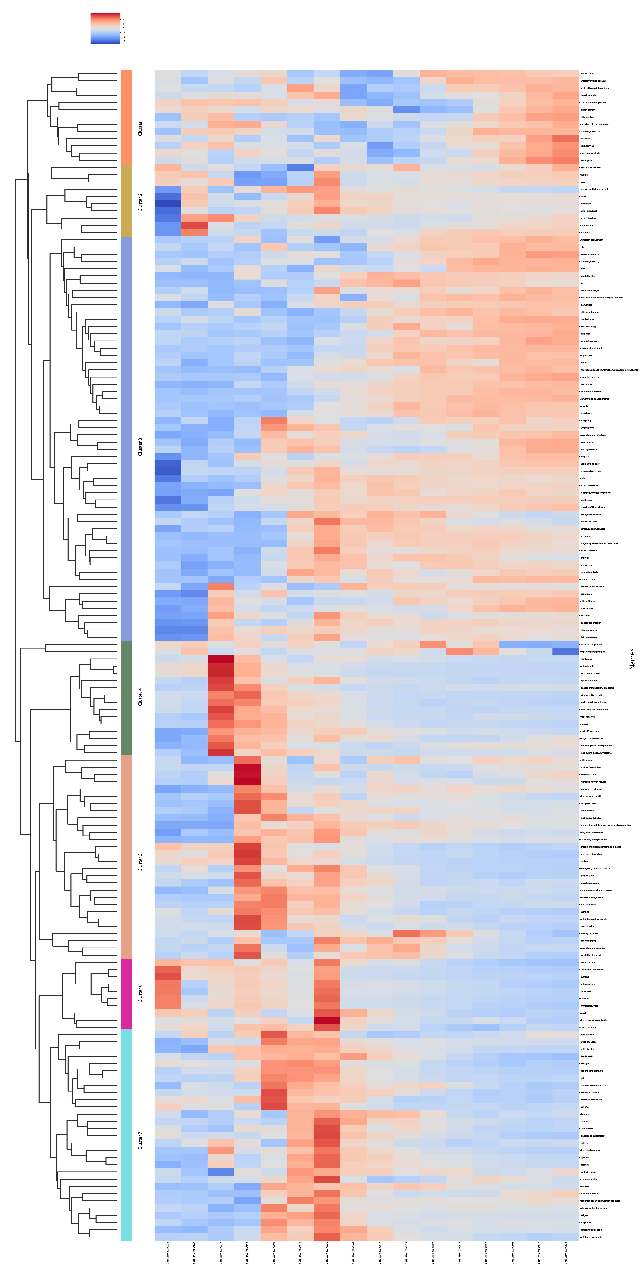


Figure S2: Heatmap of items with significant variation

**Appendix S5. List of policies**

Table S3: Names and release dates of policies listed in Table 1 in the main text

| **Cluster** | **Policy titles** | **Issuance Date** | **Relevant Content** |
| --- | --- | --- | --- |
| Patient | *Notice of the National Health Commission on Effectively Conducting Related Work at Infant Care Institutions*  *国家卫生健康委关于做好托育机构相关工作的通知* | 2020.01.28 | Children |
|  | *Notice on Effectively Conducting COVID-19 Prevention and Control Work in Elderly People*  *关于做好老年人新型冠状病毒感染肺炎疫情防控工作的通知* | 2020.01.29 | Aged patient |
|  | *Notice on Issuing the Recent Work Plan for COVID-19 Prevention and Control*  *关于印发近期防控新型冠状病毒感染的肺炎工作方案的通知* | 2020.01.28 | Aged patient |
|  | *Notice on Effectively Conducting COVID-19 Prevention and Control Work in Children and Pregnant Women*  *关于做好儿童和孕产妇新型冠状病毒感染的肺炎疫情防控工作的通知* | 2020.02.02 | Children and pregnant women |
|  | *Notice on Strengthening Treatment and Safe Assisted Delivery Services to Pregnant Women during COVID-19 Prevention and Control*  *关于加强新型冠状病毒肺炎疫情防控期间孕产妇疾病救治与安全助产工作的通知* | 2020.02.08 | Children and pregnant women |
|  | *Notice on further Effectively Conducting COVID-19 Prevention and Control Work at Medical Care and Elderly Care-Combined Institutions*  *关于进一步做好医养结合机构新冠肺炎疫情防控工作的通知* | 2020.02.18 | Aged patient |
|  | *Notice on Effectively Offering Care Services For the Elderly Relatives of Medical Staff Working at the Frontline of COVID-19 Prevention and Control*  *关于做好新冠肺炎疫情防控一线医务人员老年亲属关爱服务工作的通知* | 2020.02.22 | Aged patient |
|  | *Emergency Notice on Effectively Preventing and Controlling Clustered Outbreaks of COVID-19*  *关于切实做好新冠肺炎聚集性疫情防控工作的紧急通知* | 2020.02.26 | All |
|  | *Diagnosis and Treatment Protocol of COVID-19 (Trial Version 7)*  *新型冠状病毒肺炎诊疗方案（试行第七版）* | 2020.03.04 | Children and pregnant women |
|  | *Working Plan for Assisting and Protecting Children Without Guardianship Due to the COVID-19 Pandemic*  *因新冠肺炎疫情影响造成监护缺失儿童救助保护工作方案* | 2020.03.14 | Children |
|  | *Notice by the Joint Prevention and Control Mechanism of the State Council in Response to the Novel Coronavirus Pneumonia on further Effectively Conducting COVID-19 Prevention and Control Work for Key Locations, Organizations, and Population Groups*  *国务院应对新型冠状病毒感染肺炎疫情联防联控机制关于进一步做好重点场所重点单位重点人群新冠肺炎疫情防控相关工作的通知* | 2020.04.08 | All |
|  | *Notice on Issuing the Technical Guidelines for COVID-19 Prevention and Control Work for Key Locations, Organizations, and Population Groups*  *关于印发重点场所重点单位重点人群新冠肺炎疫情防控相关防控技术指南的通知* | 2020.04.09 | All |
|  | *Guiding Opinions by the Joint Prevention and Control Mechanism of the State Council in Response to the Novel Coronavirus Pneumonia on Effectively Conducting the Work concerning Regular COVID-19 Prevention and Control*  *国务院应对新型冠状病毒感染肺炎疫情联防联控机制关于做好新冠肺炎疫情常态化防控工作的指导意见* | 2020.05.08 | All |
|  | *Notice on Issuing the Technical Plan for COVID-19 Prevention and Control at Primary and Middle School, Nurseries, and Kindergartens*  *关于印发中小学校和托幼机构新冠肺炎疫情防控技术方案的通知* | 2020.05.08 | Children |
|  | *Notice by the Ministry of Civil Affairs, the Ministry of Housing and Urban-Rural Development, the National Health Commission, the Ministry of Emergency Management, and the State Administration for Market Regulation on Effectively Conducting Special Actions for the Construction of Service Quality of Residential Care Homes in 2020*  *民政部 住房城乡建设部 国家卫生健康委 应急管理部 市场监管总局关于做好2020年养老院服务质量建设专项行动工作的通知* | 2020.05.09 | Aged patient |
|  | *Notice by the General Office of the National Health Commission on Offering Guiding Suggestions about Nutrition and Health to Key Population Groups During the COVID-19 Pandemic*  *国家卫生健康委办公厅关于印发新冠肺炎疫情期间重点人群营养健康指导建议的通知* | 2020.05.13 | All |
|  | *Notice by the China National Committee on Aging on Effectively Offering Care Services to Elderly People During Regular COVID-19 Prevention and Control*  *全国老龄办关于在常态化疫情防控中做好老年人照顾服务工作的通知* | 2020.05.29 | Aged patient |
|  | *Notice by the General Office of the National Health Commission on further Strengthening Supervision over the Prevention and Control of Infectious Diseases at Schools*  *国家卫生健康委办公厅关于进一步加强学校传染病防控监督工作的通知* | 2020.06.01 | Children |
|  | *Notice on Issuing the Protection Guidelines for Regular COVID-19 Prevention and Control Work for Key Locations, Organizations, and Population Groups in Low-Risk Regions in the Summer (Revision)*  *关于印发低风险地区夏季重点场所重点单位重点人群新冠肺炎疫情常态化防控相关防护指南（修订版）的通知* | 2020.06.18 | All |
| Clinical Characteristics | *Notice on Issuing the Plan for COVID-19 Prevention and Control (Version 3)*  *关于印发新型冠状病毒感染的肺炎防控方案（第三版）* | 2020.01.28 | Severe symptom |
|  | *Notice on Conducting National-Level Teleconsultation Work for Severe and Critically Ill COVID-19 Patients at National Telemedicine And Connected Health Center*  *关于在国家远程医疗与互联网医学中心开展新冠肺炎重症危重症患者国家级远程会诊工作的通知* | 2020.02.01 | Severe symptom |
|  | *Notice on Issuing the Diagnosis and Treatment Protocol of COVID-19 (Trial Version 5)*  *关于印发新型冠状病毒感染的肺炎诊疗方案（试行第五版）* | 2020.02.04 | Severe symptom and asymptomatic infection |
|  | *Plan for COVID-19 Prevention and Control (Version 4)*  *新型冠状病毒肺炎防控方案（第四版）* | 2020.02.07 | Severe symptom and asymptomatic infection |
|  | *Notice on Issuing the Diagnosis and Treatment Protocol of COVID-19 (Trial Version 6)*  *关于印发新型冠状病毒肺炎诊疗方案（试行第六版）* | 2020.02.18 | Severe symptom and asymptomatic infection |
|  | *Notice on Issuing the Plan for COVID-19 Prevention and Control (Version 5)*  *关于印发新型冠状病毒肺炎防控方案（第五版）的通知* | 2020.02.21 | Severe symptom and asymptomatic infection |
|  | *Notice on Issuing the Standards of Nursing for Severe And Critically Ill COVID-19 Patients*  *关于印发新冠肺炎重型、危重型患者护理规范的通知* | 2020.03.01 | Severe symptom |
|  | *Plan for Clinical Treatment With Recovered Plasma Donated by Patients Cured of COVID-19 (Trial Version 2)*  *新冠肺炎康复者恢复期血浆临床治疗方案（试行第二版）* | 2020.03.04 | Severe symptom |
|  | *Diagnosis and Treatment Protocol of COVID-19 (Trial Version 7)*  *新型冠状病毒肺炎诊疗方案（试行第七版）* | 2020.03.04 | Severe symptom and asymptomatic infection |
|  | *Notice on Issuing the Plan for Diagnosis and Treatment of Severe And Critically Ill COVID-19 Patients (Trial Version 2)*  *关于印发新型冠状病毒肺炎重型、危重型病例诊疗方案（试行 第二版）的通知* | 2020.04.01 | Severe symptom |
|  | *Notice by the Joint Prevention and Control Mechanism of the State Council in Response to the Novel Coronavirus Pneumonia on Issuing the Management Standards for Asymptomatic COVID-19 Cases*  *国务院应对新型冠状病毒感染肺炎疫情联防联控机制关于印发新冠病毒无症状感染者管理规范的通知* | 2020.04.08 | Asymptomatic infection |
|  | *Notice on further Consolidating the Achievements and Improving the COVID-19 Prevention, Control, and Treatment Capacity of Medical Institutions*  *关于进一步巩固成果提高医疗机构新冠肺炎防控和救治能力的通知* | 2020.04.11 | Severe symptom and asymptomatic infection |
|  | *Planning for Guiding the Precise and Refined Development of Community COVID-19 Prevention and Control and Service Work*  *新冠肺炎疫情社区防控与服务工作精准化精细化指导方案* | 2020.04.16 | Asymptomatic infection |
|  | *Notice on further Effectively Conducting the Work concerning Nucleic acid Testing of the Novel Coronavirus during COVID-19*  *关于进一步做好疫情期间新冠病毒检测有关工作的通知* | 2020.04.19 | Asymptomatic infection |
|  | *Guiding Opinions by the Joint Prevention and Control Mechanism of the State Council in Response to the Novel Coronavirus Pneumonia on Effectively Conducting the Work concerning Regular COVID-19 Prevention and Control*  *国务院应对新型冠状病毒感染肺炎疫情联防联控机制关于做好新冠肺炎疫情常态化防控工作的指导意见* | 2020.05.08 | Severe symptom |
|  | *Notice on Issuing the Rehabilitation Treatment Program for COVID-19 Patients Discharged from Hospitals*  *关于印发新冠肺炎出院患者主要功能障碍康复治疗方案的通知* | 2020.05.14 | Severe symptom |
|  | *Notice on Issuing the Plan for Strengthening Public Health Capacity with Regard to Epidemic Control and Treatment*  *关于印发公共卫生防控救治能力建设方案的通知* | 2020.05.21 | Severe symptom and asymptomatic infection |
|  | *Implementing Opinions on Accelerating the Progress of Nucleic Acid Testing of the Novel Coronavirus*  *关于加快推进新冠病毒核酸检测的实施意见* | 2020.06.08 | Severe symptom and asymptomatic infection |
|  | *Notice on Issuing the Recent Work Plan for COVID-19 Prevention and Control*  *关于印发近期防控新型冠状病毒感染的肺炎工作方案的通知* | 2020.01.28 | Severe symptom |
| Antibody Test | *Diagnosis and Treatment Protocol of COVID-19 (Trial Version 7)*  *新型冠状病毒肺炎诊疗方案（试行第七版）* | 2020.03.04 | / |
|  | *Technical Guidelines for Laboratory Testing of COVID-19*  *新型冠状病毒肺炎实验室检测技术指南* | 2020.03.08 |  |
|  | *Notice on Effectively Conducting Testing of the Novel Coronavirus for Defending against Inbound Cases In Major Cities*  *关于做好防范疫情输入相关城市新型冠状病毒检测工作的通知* | 2020.04.05 |  |
|  | *Notice on Issuing the Plan for Laboratory Testing of People under Concentrated Medical Observation*  *关于印发集中医学观察人员实验室检测方案的通知* | 2020.04.06 |  |
|  | *Notice on Issuing the Plan for Offering Psychological Counseling and Social Work Services To COVID-19 Patients, Isolated People, and Their Families*  *关于印发新冠肺炎患者、隔离人员及家属心理疏导和社会工作服务方案的通知* | 2020.04.07 |  |
|  | *Notice on further Consolidating the Achievements and Improving the COVID-19 Prevention, Control, And Treatment Capacity of Medical Institutions*  *关于进一步巩固成果提高医疗机构新冠肺炎防控和救治能力的通知* | 2020.04.11 |  |
|  | *Notice on Effectively Conducting COVID-19 Testing and Health Management Service Work for People Leaving Wuhan*  *关于做好离汉人员新冠肺炎检测和健康管理服务工作的通知* | 2020.04.18 |  |
|  | *Notice on further Effectively Conducting the Work concerning Nucleic Acid Testing of the Novel Coronavirus during COVID-19*  *关于进一步做好疫情期间新冠病毒检测有关工作的通知* | 2020.04.19 |  |
|  | *Notice on Conducting Special Inspection over the Implementation of Measures for the Prevention and Control of the COVID-19 Outbreak in The Resumption of Operation and Production of Enterprises and Public Institutions*  *关于开展企事业单位复工复产疫情防控措施落实情况专项检查工作的通知* | 2020.04.27 |  |
|  | *Guiding Opinions by the Joint Prevention and Control Mechanism of the State Council in Response to the Novel Coronavirus Pneumonia on Effectively Conducting the Work concerning Regular COVID-19 Prevention and Control*  *国务院应对新型冠状病毒感染肺炎疫情联防联控机制关于做好新冠肺炎疫情常态化防控工作的指导意见* | 2020.05.08 |  |
|  | *Notice on Effectively Controlling the Quality of Nucleic Acid Testing of the Novel Coronavirus during Regular COVID-19 Prevention and Control*  *关于做好疫情常态化防控下新冠病毒核酸检测质量控制工作的通知* | 2020.06.02 |  |
|  | *Implementing Opinions on Accelerating The Progress of Nucleic Acid Testing of the Novel Coronavirus*  *关于加快推进新冠病毒核酸检测的实施意见* | 2020.06.08 |  |
|  | *Notice on further Accelerating The Capacity of Medical Institutions For Nucleic Acid Testing of the Novel Coronavirus*  *关于进一步加快提高医疗机构新冠病毒核酸检测能力的通知* | 2020.07.02 |  |
|  | *Notice on Issuing the Work Manual for Nucleic Acid Testing of the Novel Coronavirus at Medical Institutions (Trial)*  *关于印发医疗机构新型冠状病毒核酸检测工作手册（试行）的通知* | 2020.07.13 |  |
|  | *Notice on Issuing the Technical Specifications for 10-In-1 Test of the Novel Coronavirus*  *关于印发新冠病毒核酸10合1混采检测技术规范的通知* | 2020.08.19 |  |
| Chinese Medicine | *Notice by the Office of the State Administration of Traditional Chinese Medicine on Effectively Conducting the Prevention and Control of Respiratory Infectious Diseases at Traditional Chinese Medicine Hospitals*  *国家中医药管理局办公室关于做好中医医院呼吸道传染病防治工作的通知* | 2020.01.16 | / |
|  | *Notice by the Office of the State Administration of Traditional Chinese Medicine On further Effectively Conducting COVID-19 Prevention and Control With Traditional Chinese Medicine*  *国家中医药管理局办公室关于进一步做好新型冠状病毒感染的肺炎中医药防控工作的通知* | 2020.01.26 |  |
|  | *Notice on further Effectively Conducting the Treatment of COVID-19 Through Combining Traditional Chinese and Western Medicine*  *关于进一步做好新型冠状病毒感染的肺炎中西医结合救治工作的通知* | 2020.01.27 |  |
|  | *Notice on Issuing the Diagnosis and Treatment Protocol of COVID-19(Trial Version 4)*  *关于印发新型冠状病毒感染的肺炎诊疗方案(试行第四版）的通知* | 2020.01.28 |  |
|  | *Notice on Issuing the Diagnosis and Treatment Protocol of COVID-19 (Trial Version 5)*  *关于印发新型冠状病毒感染的肺炎诊疗方案（试行第五版）的通知* | 2020.02.06 |  |
|  | *Notice on Recommending the Use of “Lung Cleansing and Detoxifying Preparation” in the Treatment of COVID-19 through Combining Traditional Chinese and Western Medicine*  *关于推荐在中西医结合救治新型冠状病毒感染的肺炎中使用“清肺排毒汤”的通知* | 2020.02.07 |  |
|  | *Notice by the Office of the State Administration of Traditional Chinese Medicine on Strengthening Information Technology Support for COVID-19 Prevention and Control with Traditional Chinese Medicine*  *国家中医药管理局办公室关于加强信息化支撑新型冠状病毒肺炎疫情中医药防控工作的通知* | 2020.02.08 |  |
|  | *Notice on Issuing the Diagnosis and Treatment Protocol of COVID-19 (Trial Version 5 Revision)*  *关于印发新型冠状病毒肺炎诊疗方案（试行第五版 修正版）的通知* | 2020.02.09 |  |
|  | *Notice on Establishing a Sound Mechanism for Collaboration Between Traditional Chinese and Western Medicine in the Prevention and Control of COVID-19 and Other Infectious Diseases*  *关于在新型冠状病毒肺炎等传染病防治工作中建立健全中西医协作机制的通知* | 2020.02.12 |  |
|  | *Notice on further Strengthening the Administration of Private Medical Services and Effectively Conducting COVID-19 Prevention and Control Work*  *关于进一步加强社会办医管理做好新冠肺炎疫情防控工作的通知* | 2020.02.16 |  |
|  | *Notice on Issuing the Diagnosis and Treatment Protocol of COVID-19 (Trial Version 6)*  *关于印发新型冠状病毒肺炎诊疗方案（试行第六版）的通知* | 2020.02.19 |  |
|  | *Notice on Issuing Guiding Suggestions about the Use of Traditional Chinese Medicine for Rehabilitation in the Recovery Period of COVID-19 (Trial)*  *关于印发新型冠状病毒肺炎恢复期中医康复指导建议（试行）的通知* | 2020.02.23 |  |
|  | *Notice on Issuing the Diagnosis and Treatment Protocol of COVID-19 (Trial Version 7)*  *关于印发新型冠状病毒肺炎诊疗方案（试行第七版）的通知* | 2020.03.04 |  |
|  | *Notice on Issuing the Plan for Diagnosis and Treatment of Severe and Critically Ill COVID-19 Patients (Trial Version 2)*  *关于印发新型冠状病毒肺炎重型、危重型病例诊疗方案（试行 第二版）的通知* | 2020.04.01 |  |
|  | *Notice on Issuing the Rehabilitation Treatment Program for COVID-19 Patients Discharged from Hospitals*  *关于印发新冠肺炎出院患者主要功能障碍康复治疗方案的通知* | 2020.05.14 |  |
|  | *Notice by the Office of the State Administration of Traditional Chinese Medicine on Effectively Conducting the Work concerning Traditional Chinese Medicine During Regular COVID-19 Prevention and Control*  *国家中医药管理局办公室关于做好新冠肺炎疫情常态化防控中医药各项工作的通知* | 2020.05.15 |  |
|  | *Notice by the Office of the State Administration of Traditional Chinese Medicine on Issuing the Guidelines for The Construction And Management of the Center for Quality Control of Traditional Chinese Medicine (Trial)*  *国家中医药管理局办公室关于印发中医病案质量控制中心建设与管理指南（试行）的通知* | 2020.05.20 |  |
|  | *Notice on Issuing the Plan for Strengthening Public Health Capacity with Regard To Epidemic Control and Treatment*  *关于印发公共卫生防控救治能力建设方案的通知* | 2020.05.22 |  |
|  | *Notice by the Office of the State Administration of Traditional Chinese Medicine on further Strengthening COVID-19 Prevention and Control Work at Medical Institutions of Traditional Chinese Medicine*  *国家中医药管理局办公室关于进一步强化中医医疗机构新冠肺炎疫情防控工作的通知* | 2020.06.20 |  |
| Mental Health | *Notice on Issuing the Guiding Principles for Emergency Psychological Crisis Interventions during the COVID-19 Pandemic*  *关于印发新型冠状病毒感染的肺炎疫情紧急心理危机干预指导原则的通知* | 2020.01.27 | / |
|  | *Notice on Setting up Psychological Assistance Hotlines in Dealing With the Pandemic*  *关于设立应对疫情心理援助热线的通知* | 2020.02.02 |  |
|  | *Notice on Several Measures for Improving The Working Conditions of Frontline Medical Staff and Caring About Their Physical And Psychological Health*  *关于改善一线医务人员工作条件切实关心医务人员身心健康若干措施的通知* | 2020.02.10 |  |
|  | *Notice on Implementing Several Measures for Improving the Working Conditions of Frontline Medical Staff and Caring about Their Physical and Psychological Health*  *关于贯彻落实改善一线医务人员工作条件切实关心医务人员身心健康若干措施的通知* | 2020.02.15 |  |
|  | *Notice on Strengthening Psychological Assistance and Social Work Services in Dealing with the COVID-19 Pandemic*  *关于加强应对新冠肺炎疫情工作中心理援助与社会工作服务的通知* | 2020.03.05 |  |
|  | *Notice on Issuing the Work Plan for Psychological Counseling during the COVID-19 Pandemic*  *关于印发新冠肺炎疫情心理疏导工作方案的通知* | 2020.03.18 |  |
|  | *Notice on Issuing the Plan for Offering Psychological Counseling and Social Work Services to COVID-19 Patients, Isolated People, and Their Families*  *关于印发新冠肺炎患者、隔离人员及家属心理疏导和社会工作服务方案的通知* | 2020.04.07 |  |
|  | *Notice on Issuing the Plan for Psychological Counseling and Social Work Services for People Entering China*  *关于印发入境人员心理疏导和社会工作服务方案的通知* | 2020.04.21 |  |
|  | *Guiding Opinions by the Joint Prevention and Control Mechanism of the State Council In Response to the Novel Coronavirus Pneumonia on Effectively Conducting the Work concerning Regular COVID-19 Prevention and Control*  *国务院应对新型冠状病毒感染肺炎疫情联防联控机制关于做好新冠肺炎疫情常态化防控工作的指导意见* | 2020.05.08 |  |

**References:**

Lee, J. H., Zhang, D., Kwak, S. E., Shin, H. E., Moon, H. Y., & Song, W. (2019). Effects of Acute Aerobic Exercise on the Early Stage of Energy Metabolism Related Gene Expression in Mouse Gastrocnemius: Microarray Analysis. Exercise Science, 28(1), 49-59.

Cheng, H., Yang, X., Si, H., Saleh, A. D., Xiao, W., Coupar, J., ... & Prince, M. E. (2018). Genomic and transcriptomic characterization links cell lines with aggressive head and neck cancers. Cell reports, 25(5), 1332-1345.

Tang, Li, & Guangyuan Hu(2013). Tracing the footprint of knowledge spillover: Evidence from U.S.-China Collaboration in Nanotechnology. Journal of the American Society for Information Science and Technology, 64(9), 1791-1801

Van Grembergen, O., Bizet, M., Eric, J., Calonne, E., Putmans, P., Brohée, S., ... & Defrance, M. (2016). Portraying breast cancers with long noncoding RNAs. Science advances, 2(9), e1600220.
