# Supplementary figures and images for: Coevolution of COVID-19 research and China’s policies
Source: Health Res Policy Syst. 2021 Sep 6;19:121. doi: 10.1186/s12961-021-00770-6 (PMC8419657; doi:10.1186/s12961-021-00770-6)

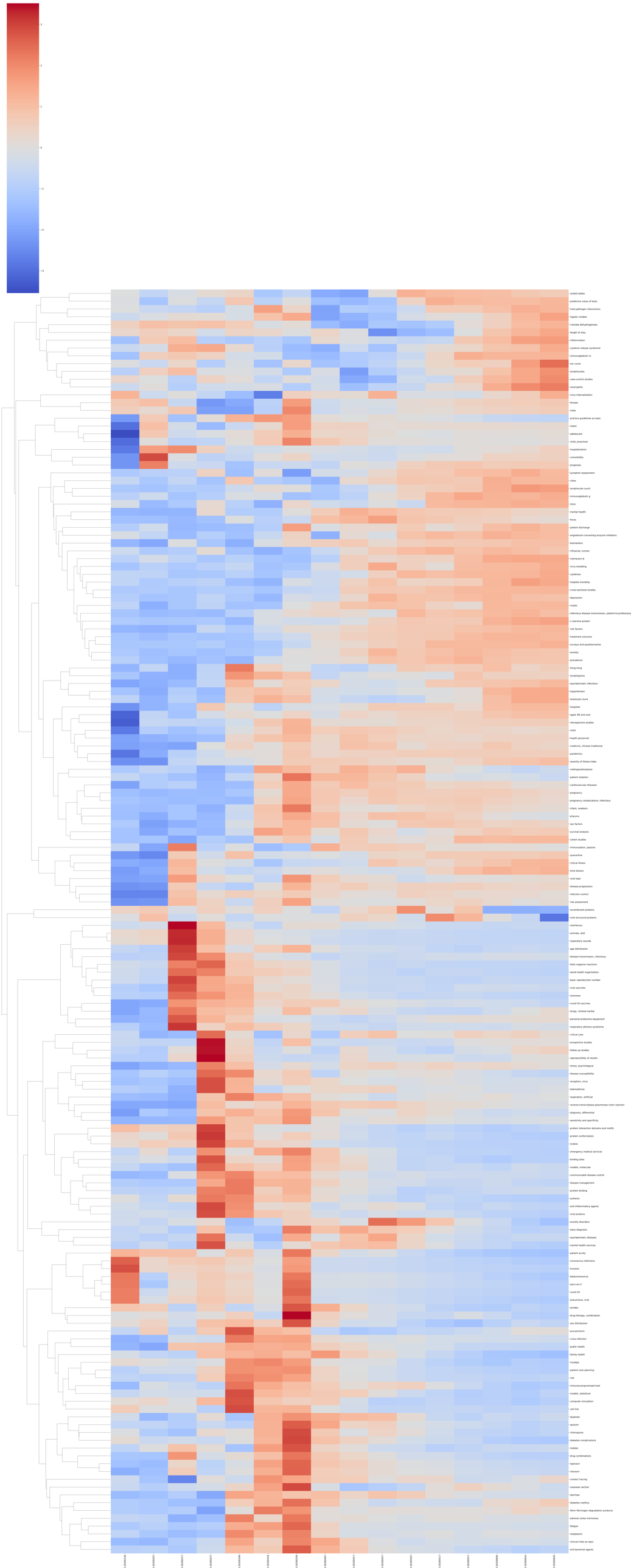

Supplement: Supplementary file 2 — Additional file 2: Heatmap of items with significant variation. [file 12961_2021_770_MOESM2_ESM.pdf]
